# Mercury Levels in Women and Children from Interior Villages in Suriname, South America

**DOI:** 10.3390/ijerph15051007

**Published:** 2018-05-17

**Authors:** Paul E. Ouboter, Gwendolyn Landburg, Gaitrie U. Satnarain, Sheryl Y. Starke, Indra Nanden, Bridget Simon-Friedt, William B. Hawkins, Robert Taylor, Maureen Y. Lichtveld, Emily Harville, Jeffrey K. Wickliffe

**Affiliations:** 1 National Zoological Collection of Suriname/Environmental Research Center (NZCS/CMO), Anton de Kom University of Suriname, Paramaribo, Suriname; p.ouboter@uvs.edu (P.E.O.); Gwendolyn.Landburg@uvs.edu (G.L.); Gaitrie.Satnarain@uvs.edu (G.U.S.); sherylstarke@yahoo.com (S.Y.S.); Indra.Asraf-Nanden@uvs.edu (I.N.); 2 Department of Global Environmental Health Sciences, Tulane University, New Orleans, LA 70112, USA; bsimon@tulane.edu (B.S.-F.); mlichtve@tulane.edu (M.Y.L.); 3 Department of Health Policy, Vanderbilt University, Nashville, TN 37203, USA; brad.hawkins@vanderbilt.edu; 4 Department of Veterinary Integrative Biosciences, Texas A&M University, College Station, TX 77843, USA; rtaylor@cvm.tamu.edu; 5 Department of Epidemiology, Tulane University, New Orleans, LA 70112, USA; eharvill@tulane.edu

**Keywords:** mercury, gold-mining, Suriname, biomarkers, fish, hair samples, CVAAS

## Abstract

Natural sources of mercury, historical gold mining, and contemporary artisanal and small-scale gold mining (ASGM) activities have led to mercury contamination in Suriname. Our primary objective was to evaluate mercury levels in hair of women and children from interior villages in Suriname where mercury levels in fish are elevated. We also estimated blood levels of mercury using an established mathematical conversion to facilitate comparison with other biomonitoring programs in the United States. Estimated levels of mercury in the blood of participants from Suriname were significantly higher than those in women from a heavy marine fish-consuming population in southeast Louisiana and estimates of the US national average. This includes women from Surinamese villages well upstream of ASGM activities. Since residents in these areas rely heavily on local fish, this is likely the source of their exposure to mercury. The levels in hair are similar to those seen in women from longitudinal studies finding neurological impairments in children exposed pre- and postnatally. Additional biomonitoring and neurodevelopmental assessments are warranted in these areas, as well as other areas of the Suriname. Mercury levels in hair (Suriname) and blood (southeast LA USA) were determined using cold vapor atomic absorption spectroscopy (CVAAS).

## 1. Introduction

Mercury is a ubiquitous environmental element that can be found in a variety of inorganic and organic chemical forms [1,2]. Mercury has long been studied as an environmental agent of public and human health concern. Large-scale historical poisonings (e.g., Minamata Bay) indicate how damaging exposure to high levels of environmental mercury can be [3,4]. In addition, there are also large longitudinal epidemiological and neurological health assessments that suggest much lower levels also adversely impact human health [5,6,7,8,9]. These studies, in addition to many others, indicate that the developing fetus is most vulnerable to the long-term, irreversible neurodevelopmental effects of exposure to environmental mercury, especially methylmercury [1,2,3,4,5,6,7,8,9,10,11,12]. Therefore, exposure to mercury remains a contemporary environmental health concern.

Exposure to mercury in any general, non-occupational population occurs mainly through the diet, and in many cases, through the consumption of fish [13,14,15,16,17,18,19,20,21,22,23,24,25]. The consumption of fish on a global scale is widespread, and fish is viewed as a healthy low-fat source of protein and dietary fatty acids [15,16,17,26,27,28,29]. However, the benefits of consuming fish must be balanced against any negative, neurodevelopmental effects that may be caused by mercury exposure. Mercury, as an element, does not degrade or breakdown in the environment, and it also tends to cycle through ecological and biological systems in various elemental and chemical (both inorganic and organic) forms [2,12].

Sources of mercury in the environment include both natural and anthropogenic. Natural off-gassing of mercury from the Earth’s crust is a primary source in the atmosphere [13]. Anthropogenic sources include mining, smelting, combustion of coal, industrial processes that require mercury, as well as historical and current operations in the artisanal and small-scale gold mining (ASGM) sector [2,13,30,31,32,33,34,35,36,37,38,39]. Mercury is transported atmospherically on a global scale, and is readily deposited through dry and wet processes [2,13,37]. In areas with high rainfall levels, wet deposition is expected to predominate.

The United States Environmental Protection Agency (USEPA) has set a daily intake rate or reference dose (RfD) for methylmercury by way of oral ingestion at 0.1 µg/kg body weight [40]. The World Health Organization (WHO) has also set a daily intake rate of methylmercury consistent with that set by the USEPA at 0.2 µg/kg of body weight [41]. The USEPA has also developed public health action levels at 1.1 µg/g of hair [40]. However, suggested public health actions and interventions in the United States are usually based on human blood Hg concentrations that tend to range from 5.8 µg/L to 10 µg/L of whole blood [40].

Heavy fish consumers, especially women who may become pregnant, or pregnant women along with their developing children, are at most risk from exposure to methylmercury. Women and children in coastal areas, where fish and seafood are major sources of food, as well as inland areas, where bodies of water provide an abundant supply of fish for personal consumption and commercial purposes, are expected to experience the highest exposures to methylmercury from fish consumption [17,18,19,24,29,42]. This is especially true where consumed or preferred fish are predatory piscivores and occupy higher trophic levels [21,37]. 

There are concerns regarding high exposures to mercury in the general population in the South American country of Suriname. A pilot study in 2005 found that 14 of 39 (36%) women evaluated had mercury levels in hair that were elevated in comparison with a US reference population [43]. However, women in this pilot study were recruited from the coastal capital of Paramaribo, and may not have included a representative number of women from interior areas of the country where exposure may be more widespread [36,37,38]. Fish is a major dietary protein source, especially in communities in the interior regions of the country where fish stocks in freshwater streams, rivers, and lakes are abundant [36,37,38]. In Suriname, ASGM is considered to be a probable source of environmental mercury in these areas, and high mercury levels in predatory fish have been found in most of the country [36,37,38]. Since many communities and villages in the interior of Suriname rely heavily on local finfish as part of their diet, hair mercury levels have been determined in women and children from several different sites. We have also estimated blood mercury levels using a well-established method to facilitate comparison to our biomonitoring program targeting coastal women in southeast Louisiana (LA), as well as data from the US Centers for Disease Control (USCDC) National Health and Nutrition Examination Survey (NHANES) [23,24,40,44,45]. We also provide results in the context of well-established action levels used in the United States and by the WHO for intervention purposes.

## 2. Materials and Methods 

### 2.1. Description of Surinamese Study Population

The communities of five villages were investigated regarding mercury levels in hair [37]. Of these five villages, four were located along the Saramacca River: Poesoegroenoe at approximately 60 km upstream of any gold mining activity, Njoeng Jacobkondre and Kwakoegron are located in the gold mining area, and Pikin Saron downstream of the gold mining. The fifth village is Brownsweg, that is located at the Brokopondo Reservoir, which is within the gold mining area. Brownsweg is further divided into subvillages, including Kadyu, Waki Basu I, Waki Basu II, Waki Basu III, Makambi, Nyun Gansee, and Biri Udu Mata. Pikin Saron is populated by indigenous or Amerindian people, and the other four villages are populated by people of African descent also known as Maroons. Research was approved and supported by the Ministry of Health of Suriname, local Surinamese communities and their leadership, the United States National Institutes of Health, and the Fogarty International Center.

A description of the research participants from Louisiana (LA) can be found in Zilversmit et al. [24]. Data from these participants is included and important for comparative purposes. Research on US subjects from LA was approved under Tulane University’s IRB Protocol #262504.

### 2.2. Mercury (Hg) Analysis in Hair

Approximately 1 g hair samples were collected to evaluate Hg levels as a biomarker of chronic exposure. Each sample was labeled and placed into sample bags for subsequent mercury analysis and transported to the NZCS/CMO laboratory for Hg analysis. Hair samples were rinsed with ethanol. Samples (0.5 g dry weight) were digested in 250 mL containers using 2.5 mL of both ultra-pure sulfuric acid (H_2_SO_4_) and nitric acid (HNO_3_) for 12 h at room temperature, and then heated to 75 °C for 1 h; 100 mL of ultra-pure deionized water was added to the digested solution with 15 mL of 5% potassium permanganate (KMnO_4_). Samples were then placed in a 95 °C water bath for 2 h and then allowed to cool. After cooling, 6 mL sodium chloride-hydroxylamine and 5 mL tin(II) chloride (SnCl_2_) was added to the hair samples. Mercury measurements were performed using a Bacharach mercury analyzer following cold vapor atomic absorption techniques [46]. For quality control and accuracy of the Hg analysis, standard Hg solutions were run on the same Hg analyzer. 

Blood Hg methods and analyses for samples from research participants in LA can be found in Zilversmit et al. [24]. CVAAS was used to determine mercury levels in whole blood samples from participants in LA [24].

### 2.3. Hair/Blood Hg Concentrations and Hazard Quotient Estimation

To facilitate comparison with mercury programs and research studies examining levels in whole blood in the United States, we converted the hair Hg levels in the Surinamese participants to blood Hg levels. Several studies have examined the relationship between hair and blood Hg levels [40]. Hair/blood Hg ratios ((µg Hg/g hair)/(µg Hg/L blood)) across these reports vary by as much as 3×, ranging from 140 to 416. Phelps et al. [47] established that the best estimate of the hair/blood Hg ratio lies between 200 and 296. The USEPA uses a hair/blood Hg ratio of 250 for conversions which is also the conversion factor that we used in this study [40]. Microsoft Excel for Windows (2013) was used for calculating blood Hg levels from hair Hg levels.

Microsoft Excel for Windows (2013) was used for estimating Hg daily doses for calculating hazard quotients [40]. Hazard quotients (HQs) were generated using the USEPA’s recommended RfD for methylmercury of 1 × 10^−1^ µg/kg/day and the WHO’s provisional tolerable weekly intake (PTWI) of methylmercury of 1.6 µg/kg/week (2.3 × 10^−1^ µg/kg/day). HQs were generated using individual body weights where possible, and a standard, conservative value of 60 kg when individual body weights were not available. The HQ calculation was simply the estimated daily dose divided by either the USEPA’s RfD or the WHO’s PTWI. Hazards were considered excessive when the HQ ≥ 1.

Fish consumption and intake rates were assessed using mercury levels in fish from previously published reports to determine the plausibility of consumption of locally harvested fish being the primary source of exposure [36,37,38]. The US Environmental Protection Agency (EPA) method referenced above was also used to then estimate expected hair mercury levels from possible intake rates, to assess and gauge such plausibility related to consumption of locally sourced fish with the reported mercury levels [40].

### 2.4. Statistical Analyses

Prism was used for all statistical analyses, as well as for generating graphs and summary statistics (version 7.03, GraphPad, LaJolla, CA, USA). Data were checked for normality prior to statistical testing, and if deemed non-normal, non-parametric methods were used for testing. Either parametric ANOVA or non-parametric Kruskal-Wallis tests were used to determine if there were statistically significant differences in hair or blood mercury levels among the sites, including subvillages within the Brownsweg village, in Suriname, and among Surinamese and US sites and data. Post hoc mean comparisons were corrected using Dunn’s test. The Wilcoxon signed rank test was used to analyze medians from Suriname villages and LA using theoretical medians, including the USEPA action level for blood Hg of 5.8 µg/L, and the NHANES level of 0.93 µg/L. Correlation testing (Spearman’s r) was performed to examine the relationship between repeat samples taken in April 2009 and August 2009 from the same participants in Brownsweg. The Wilcoxon matched pairs test was used to determine if there were significant differences between repeat samples from these participants as a means to examine any seasonal or temporal variability. Correlation testing (Spearman’s r) was performed to examine the relationship between hair Hg levels and age. Results from statistical testing were considered significant at *p* < 0.05.

## 3. Results

**Figure 1:**
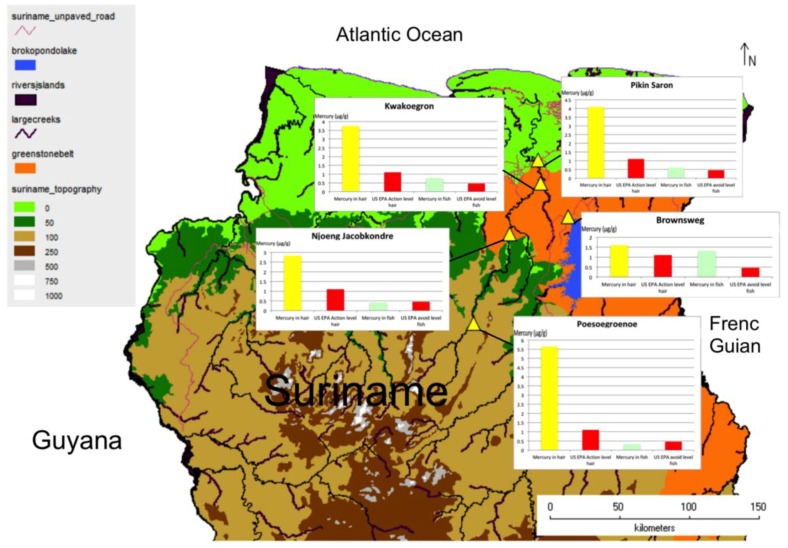
Map of the northern and central part of Suriname, showing study area and villages that have participated in the research (yellow triangles). Mercury levels in hair and fish are included to facilitate comparison. The Greenstone Belt (in orange) is the area where artisanal and small-scale gold mining (ASGM) activities are concentrated and ongoing.

Figure 1 provides a map of Suriname, and the village locations for this study. Mercury levels in hair and fish sampled from areas near each village (e.g., streams and rivers where residents collect fish for consumption) are graphically represented to facilitate comparison [36,37,38]. The region where current ASGM activities are ongoing is also indicated (Greenstone Belt), along with the prevailing wind direction.

Descriptive statistics for research participants in Suriname are presented in Table 1. All analyses were restricted to either females up to the age of 50, or those below the age of 18 (both male and female research participants in the latter group).

**Figure 2:**
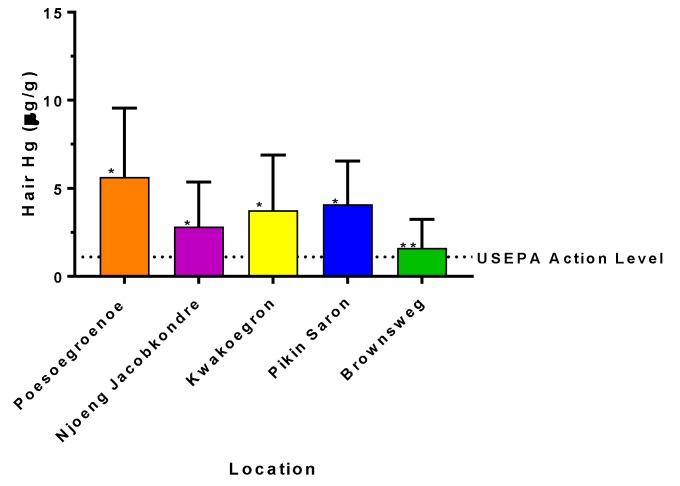
Geometric mean concentrations (±geometric standard deviation) of mercury in the hair of women and children (combined) from interior villages in Suriname. The United States Environmental Protection Agency (USEPA) action level of 1.1 µg/g is plotted for comparison. Asterisks denote significant differences at *p* < 0.05.

Hair and blood Hg data did not pass tests for normality, so all subsequent analyses used non-parametric methods. Figure 2 shows hair Hg levels for the major villages in Suriname with the USEPA’s action level. 

**Figure 3:**
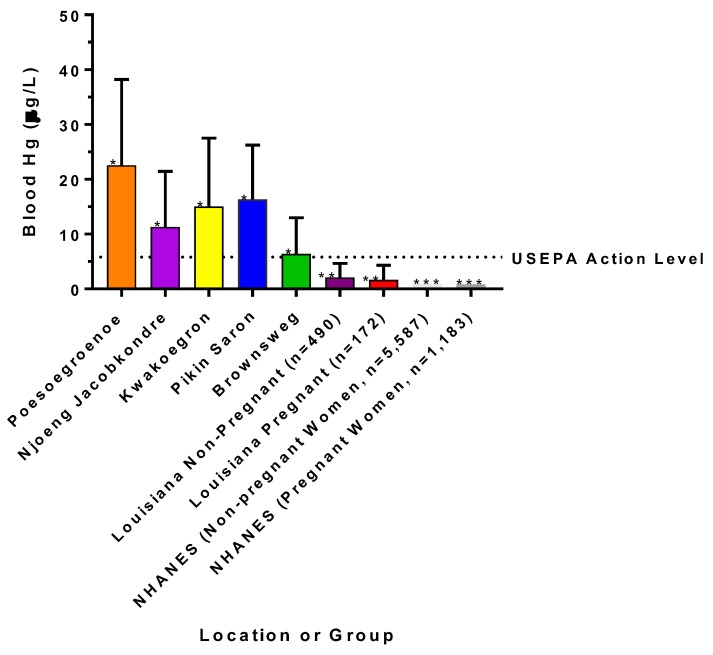
Estimated geometric mean concentrations (± geometric standard deviation) of mercury in blood of women and children (combined) from interior villages in Suriname. Geometric mean concentrations (± geometric standard deviation) of mercury in blood of women (pregnant or non-pregnant) from coastal southeast Louisiana, as well as those representing national averages from the NHANES are also presented for comparative purposes. The USEPA action level of 5.8 µg/L is plotted for comparison. Asterisks denote significant differences at *p* < 0.05.

Kruskal-Wallis (K-W) testing followed by a means comparison test indicated there were significant differences in hair Hg levels among the Surinamese villages (K-W = 69.9, *p* < 0.0001) and that Brownsweg had significantly lower levels of hair Hg than the other four villages (*p* < 0.05). Figure 3 shows blood Hg levels among Surinamese villages based on hair Hg conversions presented with US national blood Hg levels (geometric mean of the NHANES dataset) and data from research participants in southeast Louisiana.

Kruskal-Wallis testing followed by a means comparison test indicated that participants in all Surinamese villages had significantly higher levels of blood Hg (K-W = 341.2, *p* < 0.0001) than participants from southeast Louisiana (*p* < 0.0001). A Wilcoxon signed rank test using the USEPA action level of 5.8 µg Hg/L of blood as a theoretical median indicated that participants in the Surinamese villages had median levels that were significantly higher than this action level (*p* < 0.0005) and that the participants in southeast Louisiana had median levels that were significantly lower than this action level (*p* < 0.0001). Blood Hg levels did not differ among non-pregnant and pregnant women from the LA cohort (*p* < 0.16). A Wilcoxon signed rank test using the NHANES level of 0.93 µg Hg/L of blood as a theoretical median indicated that participants from LA had significantly higher median levels of blood Hg than the US national average (*p* < 0.0001).

Repeat sampling of participants in Brownsweg in April 2009 and August 2009 resulted in 125 matched pairs. Correlation analysis indicated that hair Hg levels were highly correlated (Spearman’s r = 0.79, *p* < 0.0001), and the Wilcoxon matched pairs signed rank test indicated that levels from these two time points did not significantly differ (*p* < 0.38, median difference of −0.05 µg Hg/g hair).

Kruskal-Wallis testing, followed by a means comparison test, indicated that only the subvillages of Makambi and Biri Udu Mata among all subvillages in Brownsweg had hair Hg levels that significantly differed from one another (K-W = 24.7, *p* < 0.0004).

**Figure 4:**
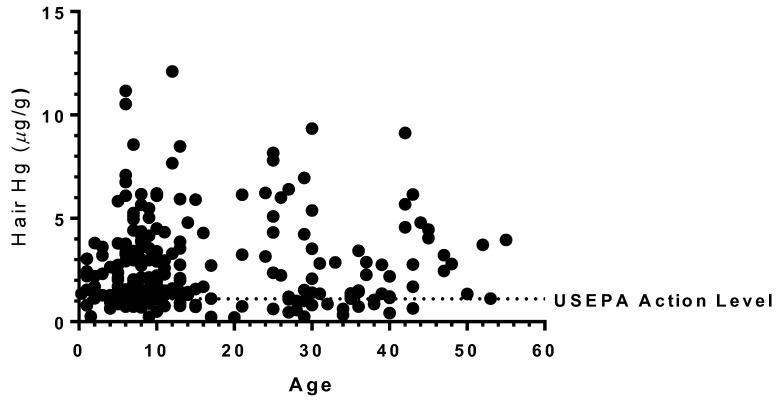
Scatterplot representing levels of mercury in hair of research participants and age. The USEPA action level of 1.1 µg/g is plotted for comparison. No significant correlation exists between hair mercury levels and age.

Correlation analysis of age and hair Hg levels with 230 matched pairs indicated no significant association between these two variables (Spearman’s r = −0.03, *p* < 0.61). Figure 4 shows a scatterplot of age and hair Hg levels.

Table 2 presents percentages of research participants in villages and subvillages in Suriname where estimated blood Hg levels exceed US action levels (5.8 µg/L or 10 µg/L). Table 2 also presents the percentages of research participants that, following estimation of daily Hg doses using either individual body mass and/or a median body mass of 60 kg, have HQs ≥ 1 using the USEPA RfD (1 × 10^−1^ µg/kg/day) or the WHO PTWI (1.6 µg/kg/week). HQs ≥ 1 for non-cancer health risks are usually used in the US as a trigger for action or intervention. Data from LA participants are included for comparison.

Table 3 represents expected or predicted hair mercury levels in average individuals from either Poesoegroenoe or Brownsweg based on different intake rates of locally harvested fish. These two villages were selected for this exercise because they represent areas with the highest levels of mercury in hair and lowest levels of mercury in fish (Poesoegroenoe, 5.6 µg/g hair, 0.3 µg/g fish) or the lowest levels of mercury in hair and highest levels of mercury in fish (Brownsweg, 1.6 µg/g hair, 1.3 µg/g fish) [36,37,38]. Residents in Poesoegroenoe rely almost exclusively on locally harvested fish as their primary source of dietary protein, while residents in Brownsweg eat much less locally harvested fish and rely more on chicken and marine fish imported from the coastal city and capital Paramaribo. Based on reasonable consumption scenarios and intake rates, the predicted hair mercury levels are consistent with those determined by the laboratory analyses, and the dietary or fish-consuming behaviors in these two villages, and likely the other villages as well. This is further support, albeit indirect, that the most likely source of exposure to mercury in these interior villages is from consuming contaminated freshwater fish that is locally harvested.

## 4. Discussion

### 4.1. Possible Sources of Exposure to Hg in Suriname

Consumption of locally harvested fish is a probable source of exposure to Hg in the participants in these Surinamese villages [32,36,37,38]. However, questions remain as to the ultimate source(s) of Hg in the environment in these interior areas. Results show high levels of Hg in participants from most of the villages examined. Ouboter et al. [37] have reported the highest levels of Hg in predatory fish in the Brokopondo Reservoir. However, mercury levels in the population of the village, Brownsweg, and its subvillages on the shore of this reservoir are relatively low. Approximately 5% of the fish eaten in Brownsweg originate from the reservoir, as most of the fish eaten is imported from the capital, Paramaribo [36]. Brownsweg is easily accessible from Paramaribo by road. Of the subvillages of Brownsweg, participants from Biri Udu Mata had the highest mercury levels in the population. This is the subvillage closest to and bordering the Brokopondo Reservoir. We suspect that more fish from the lake are consumed here, but this cannot be verified at this point because dietary data have been aggregated for the entire village of Brownsweg.

Mercury levels are highest in participants from Poesoegroenoe. This village is upstream of the active gold mining. Mercury levels in predatory fish here are elevated, but on average, they are below the EPA standard for human consumption [37]. However, Poesoegroenoe was only accessible by airplane or through a 2 day boat trip at the time the field research was conducted. At that time, the population of Poesoegroenoe was almost completely dependent on local fish as a source of protein [38]. Due to the high rate of fish consumption, including many favored predatory fish, Hg levels in the population of Poesoegroenoe are substantially elevated. It should be emphasized that although Poesoegroenoe is upstream of any active gold mining, with regard to the prevailing wind direction from the northeast, it is downwind of the gold mining. It is plausible that Hg used in the active gold mining areas evaporates, as Hg is quite volatile, and is then transported by the prevailing winds and deposited downwind to the southwest, where mountain ranges are dominant, including in the drainage area of the upper Saramacca River where Poesoegroenoe is located [37]. 

### 4.2. Possible Health Implications Based on Previous Research

The adverse effects of Hg on neurodevelopment and neurological function have been well-documented and described from relatively high-dose exposures occurring during gestation that represent effectively what could be categorized as environmental poisonings. These include the case studies illustrating the disastrous, devastating impacts in Minamata Bay, Japan, and to a lesser extent, the neurological impairments resulting from the inadvertent use of Hg-contaminated wheat in the country of Iraq [3,4]. 

There are also two large longitudinal cohort studies that have been conducted in the Seychelles in the Indian Ocean, and the Faroe Islands in the North Atlantic Ocean, along with a smaller study in New Zealand examining neurodevelopmental impacts of relatively high levels of mercury [5,6,7,8,9,18,19,29,48,49]. The studies in the Faroe Islands and New Zealand both found slight but significantly decreased performance on neuropsychological tests but not neurophysiological tests [5,6,7,8,9]. The study in New Zealand also reported a higher frequency of poor birth outcomes in their mercury-exposed group, as well as significant influences of ethnicity and social class on neuropsychological test performance [5]. Methylmercury levels in the New Zealand study still accounted for a small but significant influence on neuropsychological outcomes [5]. The study in the Seychelles found significant decrements in neuropsychological performance in an initial pilot study, but in a much larger main study, in which many confounders revealed in the pilot study were controlled for, they found no effect of methylmercury on neurodevelopment [19,29,48,49]. The apparent lack of concordance, especially between the two large island-nation studies, is not well understood, but it has been suggested that dietary differences and sources of mercury may, in part, account for those [18,19,29,50]. A principal source of mercury exposure in the Faroe Islands has been through maternal consumption of pilot whale meat and blubber in addition to finfish, while mercury exposure in the Seychelles has been through maternal consumption of finfish [18,29,49]. Researchers conducting the Seychelles study suggest that heavy fish consumption in their cohort not only results in relatively high levels of mercury exposure, but also in high levels of omega-three fatty acids [29]. Because emerging evidence suggests that omega-three fatty acids are neuroprotective, including during fetal development, they postulate that this, in part, may explain the different outcomes between these two large studies [19,29,49]. If this explanation is eventually borne out by additional research, the benefits of a diet high in finfish that are rich in omega-3 fatty acids may outweigh the health costs of consuming mercury in those same finfish [51].

At present, no systematically collected data or studies of neurodevelopment or performance on neuropsychological and neurophysiological tests are available for evaluation in Suriname. This research group, which is a partnership between investigators in Suriname and the United States, is in the process of addressing that shortcoming by measuring neurodevelopmental progress from birth through 4–5 years of age in an ongoing longitudinal cohort study of 1000 mother-child dyads. In addition, both blood (maternal and cord) and hair mercury levels are being determined in participants in this same cohort. Systematic surveys of fish consumption, including intake rates as well as species and preferences of species consumed, are being collected to better inform assessment of health risk, and if and where necessary, facilitate the development of consumption advisories tailored to communities based on literacy levels using a culturally competent approach. Based on the previous cohort studies, it is also important that other dietary components be measured, such as omega-three fatty acids both in consumed finfish and in human participants. There is also emerging evidence that selenium may improve pediatric cognitive function, as well as protect against methylmercury toxicity [52,53,54]. Marine finfish and shellfish are excellent sources of dietary selenium which may also explain differences between the two large cohort studies, but it is not clear if freshwater finfish in Suriname are either a good source of selenium or omega-three fatty acids. Addressing these gaps will allow for a more comprehensive understanding of the effects of exposure to relatively high levels of mercury among the interior communities in Suriname, and possibly elsewhere, where similar environmental, dietary, and cultural practices, behaviors, and conditions exist.

## 5. Conclusions

Despite decades of research, the effects of mercury on neurodevelopment in children below those of obvious clinical toxicity (e.g., Minamata), remain a challenge to unequivocally define. This is, in large part, because neurodevelopment is an inherently complex process influenced by a number of physical, chemical, and social factors. Relatively high levels of mercury have been found in hair samples from women and children in interior areas of Suriname, as reported in this study. At present, these levels are similar to those found in previous cohort studies that have found negative neurodevelopmental impacts on children exposed during gestation and possibly during early childhood [5,6,7,8,9]. Additional research already underway in this study will help determine if there are any neurodevelopmental effects resulting from such exposures in these culturally diverse and unique communities, in comparison to those that have been examined in previous work. This will add to the available evidence base regarding the effects of dietary methylmercury attributable to the intake of finfish and ultimately better inform public health practice.

## Figures and Tables

**Table 1:** Levels of mercury in hair and estimated levels in blood from women and children from interior villages in Suriname. Average age and body mass are reported as well.

Village (or Subvillage)	Geometric Mean Hair Hg Level in µg/g (SD ^a^)	Geometric Mean Blood Hg Level in µg/L (SD ^a^)	Arithmetic Mean Age in Years (SD ^b^)	Arithmetic Mean Body Mass in kg (SD ^b^)
Kwakoegron (*n* = 26)	3.75 (1.83)	15.0 (1.83)	24.5 (18.0)	59.9 (30.4)
Pikin Saron (*n* = 16)	4.09 (1.60)	16.4 (1.60)	21.6 (13.6)	52.4 (24.0)
Poesoegroenoe (*n* = 13)	5.64 (1.69)	22.6 (1.69)	9.9 (6.5)	N/A ^d^
Njoeng Jacobkondre (*n* = 16)	2.83 (1.89)	11.3 (1.90)	12.4 (9.6)	N/A ^d^
Brownsweg (*n* = 161)	1.60 (2.02)	6.4 (2.03)	15.8 (12.8)	38.4 (22.8)
Kadyu ^c^ (*n* = 32)	1.77 (1.94)	7.1 (1.94)	15.0 (12.5)	38.4 (25.6)
Waki Basu I ^c^ (*n* = 21)	1.53 (2.04)	6.1 (2.04)	17.9 (14.5)	42.0 (21.4)
Waki Basu II ^c^ (*n* = 15)	1.87 (2.09)	7.5 (2.09)	14.6 (11.9)	34.8 (19.7)
Waki Basu III ^c^ (*n* = 7)	1.76 (1.40)	7.0 (1.40)	19.9 (19.7)	35.9 (20.9)
Makambi ^c^ (*n* = 33)	1.09 (1.80)	4.4 (1.82)	17.0 (12.11)	41.2 (22.3)
Nyun Gansee ^c^ (*n* = 34)	1.50 (2.18)	6.0 (2.18)	14.0 (11.8)	31.1 (21.9)
Biri Udu Mata ^c^ (*n* = 20)	2.69 (1.72)	10.7 (1.73)	15.0 (13.2)	42.6 (25.8)

^a^ SD—geometric standard deviation; ^b^ SD—arithmetic standard deviation; ^c^ Subvillage of Brownsweg village; ^d^ Not available as data was not collected.

**Table 2:** Percentages of participants with estimated blood levels exceeding safe thresholds and hazard quotients according to US Environmental Protection Agency USEPA or WHO criteria.

Village (or Sub-Village)	Blood Hg > 5.8 µg/L	Blood Hg > 10 µg/L	HQ ^a^ > 1 (Individual Body Mass, USEPA RfD ^b^)	HQ > 1 (60 kg Body Mass, USEPA RfD)	HQ > 1 (60 kg Body Mass, WHO PTWI ^c^)
Kwakoegron	88.5%	80.8%	95.8%	100%	80.8%
Pikin Saron	100%	81.3%	100%	100%	81.3%
Poesoegroenoe	100%	92.3%	N/A ^e^	100%	92.3%
Njoeng Jacobkondre	81.3%	62.5%	N/A ^e^	87.5%	68.8%
Brownsweg	56.1%	27.4%	see sub-villages below	76.2%	29.9%
Kadyu ^d^	62.5%	31.3%	87.5%	84.4%	31.3%
Waki Basu I ^d^	57.1%	23.8%	81.0%	81.0%	23.8%
Waki Basu II ^d^	66.7%	46.7%	93.3%	73.3%	53.3%
Waki Basu III ^d^	57.1%	14.3%	100.0%	100.0%	28.6%
Makambi ^d^	29.0%	3.2%	81.8%	64.5%	6.5%
Nyun Gansee ^d^	57.6%	24.2%	84.6%	69.7%	27.3%
Biri Udu Mata ^d^	85.0%	65.0%	87.5%	95.0%	65.0%
Southeast LA (Non-pregnant Women)	11.3%	3.7%	N/A	14.8%	2.5%
Southeast LA (Pregnant Women)	11.4%	4.0%	N/A	13.6%	2.3%

^a^ HQ = hazard quotient calculated as the estimated daily dose of Hg divided by the USEPA Reference Dose (RfD) or WHO Provisional Tolerable Weekly Intake (PTWI); ^b^ USEPA RfD for methylmercury of 1 × 10^−1^ µg/kg/day; ^c^ WHO PTWI of 1.6 µg/kg/week; ^d^ Subvillages in Brownsweg; ^e^ Not available as data was not collected.

**Table 3:** Predicted mercury levels in hair of in average individuals from the interior villages of Poesoegroenoe and Brownsweg. Predicted levels of hair mercury were estimated using fish consumption scenarios and plausible intake rates, as well as average mercury levels in freshwater fish collected from streams and rivers in the areas surrounding the villages [36,37,38]. Modeled scenarios are based on the number of 4 ounce or 114 g servings (svg (s)) consumed per week. Hazard quotients are again provided to demonstrate under which consumption scenarios the intake rates would result in unacceptably high neurodevelopmental health risks. This exercise does support that for the heavy fish consuming village of Poesoegroenoe that a single 4 ounce or 114 g serving per day (7 svgs/week) of locally harvested fish could result in the observed mercury levels in hair. Consistent with the dietary behaviors reported from the village of Brownsweg consuming a much lower amount of locally harvested fish (~0.5 svgs/week or 51.0 g/week) can explain their lower levels of mercury in hair. IRs-intake rates, svg(s)-serving(s).

**Poesoegroenoe**				
**IRs**	**Ounces/Svg**	**Grams/Svg**	**HQ**	**Predicted Hair Hg Level (µg/g)**
1 svg/week	4	113.4	0.69	0.8
2 svgs/week	8	226.8	1.39	1.6
3 svgs/week	12	340.2	2.08	2.5
4 svgs/week	16	453.6	2.78	3.3
7 svgs/week	28	793.8	4.86	5.8
**Brownsweg**				
**IRs**	**Ounces/Svg**	**Grams/Svg**	**HQ**	**Predicted Hair Hg Level (µg/g)**
1 svg/week	4	113.4	3.01	3.6
2 svgs/week	8	226.8	6.02	7.1
3 svgs/week	12	340.2	9.03	10.7
4 svgs/week	16	453.6	12.03	14.3
7 svgs/week	28	793.8	21.06	25.0
~0.5 svgs/week	1.8	51.0	1.35	1.6
